# Bariatric surgery-induced gut microbiota remodeling and innate immune activation: implications for gastrointestinal cancer immunotherapy

**DOI:** 10.3389/fimmu.2026.2004691

**Published:** 2026-10-27

**Authors:** Bingbing Shen, Yi Luo, Xinghong Tang, Zhongbao Hu, Qiujing He, Lun Wang

**Affiliations:** 1 Department of General Surgery, Affiliated Hospital of Zunyi Medical University, Zunyi, China; 2 Department of Thyroid and Breast Surgery, Affiliated Hospital of Zunyi Medical University, Zunyi, China; 3 Department of General Surgery, Nayong County People’s Hospital, Bijie, China; 4 Department of Gastrointestinal Surgery, Affiliated Hospital of Zunyi Medical University, Zunyi, China; 5 Guizhou Provincial Key Laboratory of Digestive System Diseases, Zunyi, China

**Keywords:** bariatric surgery, bile acids, GI cancers, gut microbiota, immunotherapy, innate immunity

## Abstract

Bariatric and metabolic surgery (MBS)—primarily sleeve gastrectomy and Roux-en-Y gastric bypass—provides a unique human model for evaluating how profound immunometabolic rewiring alters cancer susceptibility and therapeutic responsiveness. Beyond weight loss, post-surgical anatomical reconfiguration permanently reshapes the gut microbial ecosystem, alters enterohepatic bile acid circulation, and attenuates systemic meta-inflammation. This review synthesizes emerging evidence linking post-bariatric gut microbiota remodeling to innate immune activation, with a specific focus on its translational potential in gastrointestinal (GI) cancer immunotherapy. Mechanistically, post-operative shifts in secondary bile acids, short-chain fatty acids, and microbial pattern-associated molecular patterns (PAMPs) act through key innate sensors—including FXR, TGR5, AhR, and the cGAS-STING pathway—to reprogram tumor-associated macrophages from an M2-like immunosuppressive state toward an anti-tumor phenotype. These cellular adjustments promote dendritic cell priming, stimulate type I interferon production, and enhance CXCL11-dependent cytotoxic lymphocyte recruitment, thereby remodeling “cold” gastrointestinal tumor microenvironments. By deconstructing the multi-layered crosstalk across the gut–liver and gut–tumor axes, we outline a translational framework for leveraging non-surgical surrogates—including defined microbial consortia, engineered postbiotics, and targeted pattern-recognition receptor agonists—to overcome primary resistance to immune checkpoint inhibitors in gastrointestinal malignancies.

## 1 Introduction

Obesity has become a global health emergency and is now firmly established as a modifiable driver of more than a dozen solid tumours, including malignancies of the gastrointestinal (GI) tract (1). Among the metabolic interventions available, bariatric and metabolic surgery (MBS)—most commonly sleeve gastrectomy (SG) and Roux-en-Y gastric bypass (RYGB)—remains the most effective and durable option for sustained weight loss and remission of type 2 diabetes (2). A growing body of work indicates that the surgical remodeling of the GI tract reconfigures the gut microbiota, shifts bile acid metabolism, attenuates chronic low-grade endotoxaemia and reshapes innate immune signaling (3–5). These post-surgical changes are increasingly being explored as substrates that could be harnessed to improve cancer therapy, particularly immune checkpoint inhibitor (ICI) responsiveness, in GI cancers (6–8).

The gut microbiota and the innate immune system are deeply intertwined at the mucosal interface, where pattern-recognition receptors, antigen-presenting cells and microbial metabolites collectively set the threshold for adaptive immunity (1, 9). In obesity, this interface is destabilized: dysbiosis, impaired barrier integrity and myeloid-derived inflammation establish a permissive environment for tumor initiation and progression in the GI tract (1, 10). Because bariatric surgery simultaneously corrects several of these derangements, it provides a useful natural experiment in which the immune-metabolic consequences of microbiota remodeling can be linked to cancer-relevant endpoints (3, 6).

Our study synthesizes recent evidence on how bariatric surgery restructures the gut microbiome and the innate immune compartment, how these changes intersect with bile acid and microbial metabolite signaling, and what the resulting implications are for immunotherapy of gastrointestinal cancers—including hepatocellular carcinoma (HCC), colorectal cancer (CRC), gastric and esophageal cancer, and pancreatic ductal adenocarcinoma (PDAC) (6, 11, 12). We focus on mechanistic findings that can be translated into precision adjuncts to current immune checkpoint regimens. Overall, bariatric surgery converts an immunosuppressive microbial and metabolic state into an immunostimulatory and pro-resolving environment. The interconnected cascade—spanning anatomical alteration, microbiota and bile acid remodeling, innate immune receptor engagement, and tumor microenvironment reprogramming—is illustrated in **Figure 1**.

**Figure 1:**
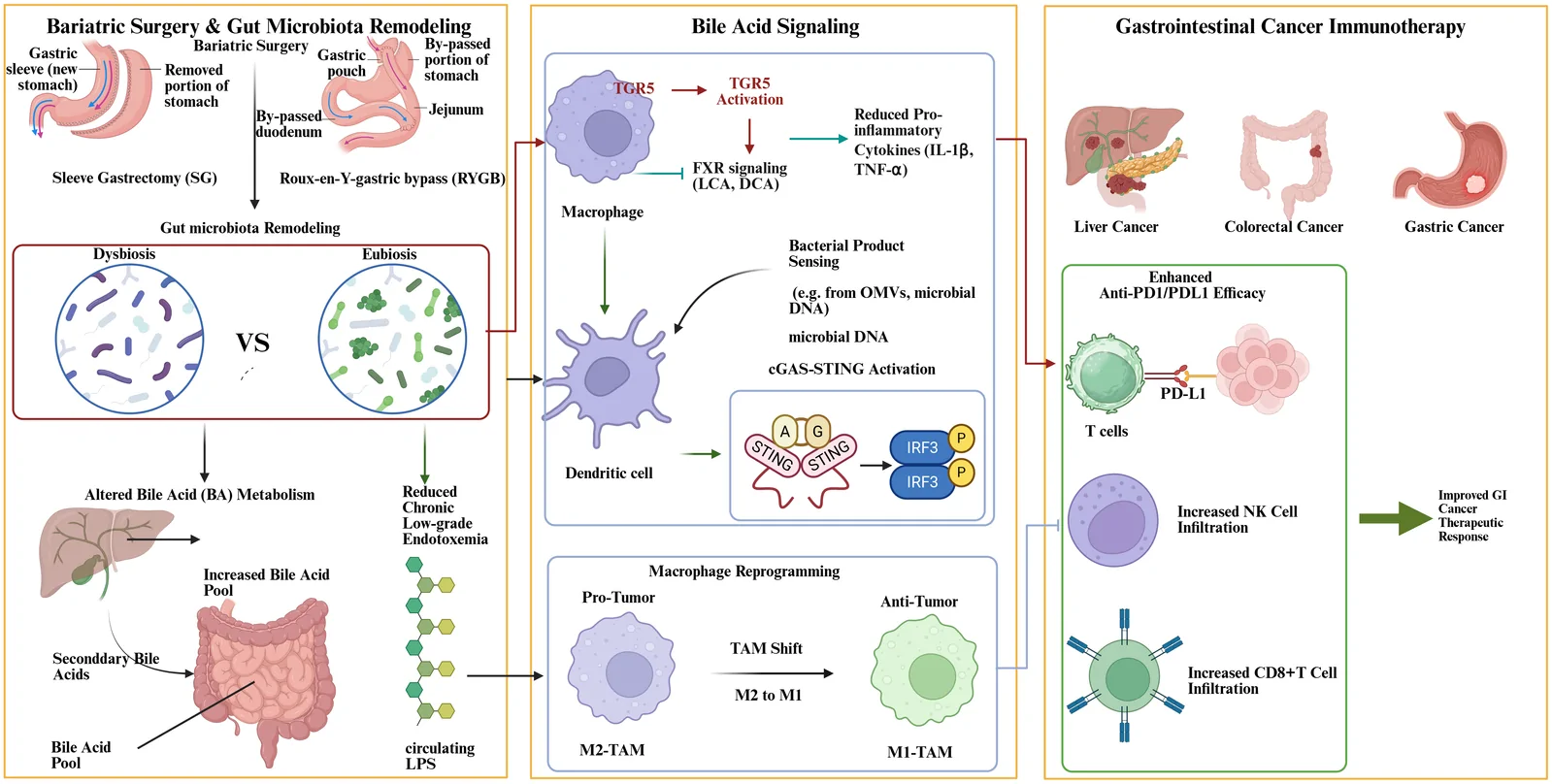
Mechanistic framework linking bariatric surgery–induced gut microbiota remodeling and innate immune activation to enhanced immune checkpoint inhibitor (ICI) efficacy in gastrointestinal cancers. SG, sleeve gastrectomy; RYGB, Roux-en-Y gastric bypass; BA, bile acid; LPS, lipopolysaccharide; TGR5, Takeda G protein-coupled receptor 5; FXR, farnesoid X receptor; LCA, lithocholic acid; DCA, deoxycholic acid; OMVs, outer membrane vesicles; cGAS-STING, cyclic GMP-AMP synthase–stimulator of interferon genes; IRF3, interferon regulatory factor 3; TAM, tumour-associated macrophage; PD-1/PD-L1, programed cell death protein 1/programed death-ligand 1; NK, natural killer; CD8^+^ T, CD8-positive T cell.

## 2 Gut microbiota remodeling after bariatric surgery

### 2.1 Procedure-dependent compositional shifts

Roux-en-Y gastric bypass and sleeve gastrectomy differ substantially in their anatomical and physiological consequences, and these differences are reflected in distinct microbial signatures (13, 14). Longitudinal 16S rRNA surveys tracking patients before and after surgery have shown that RYGB produces a larger and more durable shift in alpha- and beta-diversity than SG, with enrichment of facultative anaerobes such as Escherichia coli, Akkermansia muciniphila, Streptococcus and Veillonella, and contraction of Faecalibacterium and Romboutsia (15, 16). In a multicenter randomised comparison of RYGB and one-anastomosis gastric bypass (OAGB), microbial beta-diversity diverged significantly from baseline at 24 months even though anthropometric improvement was comparable, suggesting that procedure-specific anatomical changes—rather than weight loss per se—drive much of the taxonomic reconfiguration (13). SG also enriches oral-associated taxa in the distal gut, a phenomenon described as a canonical oral-to-gut strain sharing signature that correlates with hepatic and glycaemic remodeling after RYGB (17).

Compositional changes are detectable within weeks and persist for at least two years, with parallel shifts in the host faecal proteome and metaproteome indicating functional adaptation of the microbiota rather than transient perturbation (15). Microbial diversity also rises in the oral cavity after SG, with concurrent increases in periodontal taxa such as Actinomyces, Rothia and Streptococcus that may, in part, be explained by post-operative dietary changes and improved oral hygiene behavior (18, 19). Together, these observations support the view that bariatric surgery produces a deeply reconfigured, oligoclonal and procedure-defined microbial ecosystem rather than a partial or reversible shift (14, 20). Ultimately, surgical intervention converts the native dysbiotic architecture into a deeply reconfigured, oligoclonal, and procedure-defined ecosystem.

### 2.2 Functional and metabolic signatures

Functional metagenomic and metabolomic analyses indicate that bariatric surgery re-programes microbial pathways related to bile acid transformation, short-chain fatty acid (SCFA) production, amino acid catabolism and lipopolysaccharide (LPS) biosynthesis (21). A multi-omics comparison of SG with very-low-energy diet-induced weight loss demonstrated that RYGB uniquely enriches indoxyl sulphate and related tryptophan derivatives, even after matching for short-term weight loss, implicating the rerouted enterohepatic circulation rather than caloric restriction as the dominant driver of metabolic re-wiring (21). In mice, SG increases bile acid pools, alters hepatic bile acid synthesis enzymes such as Cyp8b1, and reshapes microbial bile acid-metabolising genes; faecal microbiota transplantation from SG donors transfers these phenotypes to naïve recipients (22, 23).

Metabolomic surveys of post-bariatric cohorts further show that 12 months after surgery, microbial gene richness and pathways for fermentation, methanogenesis and butyrate synthesis correlate with sustained type 2 diabetes remission, whereas early shifts in SCFA-producing taxa predict the trajectory of glycaemic improvement (24, 25). Predictive signatures of weight loss and fat mass reduction can be detected in the pre-operative microbiome: enrichment of Akkermansia and Bifidobacterium, together with adherence to a Mediterranean-style diet, is associated with greater long-term fat mass loss up to 24 months after bariatric surgery (26, 27). These findings suggest that microbial functional features both reflect and contribute to the metabolic benefits of bariatric surgery (2, 5). Metabolic shifts confirm that post-surgical microecology acts not merely as a passive readout, but as an active functional executor of host metabolic recovery.

### 2.3 Stability and inter-individual heterogeneity

Notwithstanding the consistency of the average signal, the post-surgical microbiome is highly individualised. Two-year metaproteomic follow-up shows that the magnitude of microbial reconfiguration, not its direction, is the principal predictor of metabolic benefit, and that pre-surgical microbial diversity explains a sizeable fraction of weight loss heterogeneity after RYGB (15, 28). Conversely, weight regain after bariatric surgery is associated with a partial reversion of the microbiota towards a baseline-like profile, although structural differences persist for years (29). These observations imply that durable metabolic benefit, and any downstream immune benefit, requires sustained perturbation of the host–microbiota interface rather than a single bolus of microbiota reconfiguration (2, 30). In other words, long-term systemic effects seem to require a lasting reset of the host–microbe relationship, not a temporary shift in the microbial community.

## 3 Innate immune activation following bariatric surgery

### 3.1 Intestinal barrier and endotoxaemia

A core metabolic effect of bariatric surgery is the reduction of obesity-associated endotoxaemia. Pre-operative obesity is characterized by impaired tight-junction integrity, bacterial translocation and elevated systemic LPS, which together drive Toll-like receptor 4 (TLR4)–dependent myeloid activation and meta-inflammation (31, 32). Bariatric surgery partially restores barrier function: in patients with severe obesity, post-operative reductions in systemic inflammatory markers coincide with enrichment of mucin-degrading and SCFA-producing taxa that support epithelial homeostasis (5, 15). FMT from post-bariatric donors into germ-free or microbiota-depleted recipients is sufficient to transfer several of these barrier-protective effects, including the attenuation of hepatic steatosis and innate immune cell infiltration in models of metabolic dysfunction-associated steatotic liver disease (MASLD) (3, 4).

Mechanistically, TLR4 and its adaptor myeloid differentiation factor 88 (MyD88) are required for the full metabolic benefit of gastric bypass in mice, and obese Tlr4-deficient animals show blunted weight loss and insulin sensitisation after surgery, suggesting that innate sensing of post-surgical microbial reconfiguration is not simply a side-effect but an active contributor to the metabolic response (33). Constitutive epithelial TLR4 activation, however, can drive duodenal adenoma formation in a microbiota- and bile-acid-dependent fashion, underlining the dual nature of innate sensing in the GI tract (34). This dual role emphasizes that post-surgical innate sensing operates as a tightly regulated balancing act between mucosal repair and homeostatic surveillance.

### 3.2 Mucosal myeloid and innate lymphoid compartments

Bariatric surgery reshapes the intestinal innate immune compartment beyond the classical monocyte–macrophage axis. In murine models, RYGB alters the macrophage and dendritic cell transcriptional landscape in the lamina propria and promotes an anti-inflammatory phenotype characterized by reduced IL-1β and elevated IL-10 production (32, 35). In humans, prospective studies report a contraction of pro-inflammatory macrophage gene signatures in peripheral leukocytes six months after SG or RYGB, with parallel increases in regulatory dendritic cell and innate lymphoid cell populations (36, 37). Mucosal innate lymphoid cells (ILC3s) are particularly responsive to surgery-related changes in secondary bile acids and tryptophan metabolites, and reductions in tissue-resident ILC3s after surgery can transiently destabilise epithelial defence until the microbiota is fully reconfigured (38, 39).

The oral-gut axis contributes additional myeloid signals: transfer of oral taxa into the distal gut after SG or RYGB is associated with altered hepatic and glycaemic endpoints, suggesting that oral-derived innate ligands—through pattern recognition receptors in the gut mucosa—contribute to systemic immunometabolic re-wiring (17). In adolescent cohorts, post-VSG stool transfer into germ-free mice reproduces a Th17-skewed, potentially inflammatory phenotype with elevated calprotectin, indicating that the immune consequences of bariatric surgery may not be uniformly protective and depend on the developmental window in which surgery is performed (40). Such divergence demonstrates that the immunological outcome of bariatric surgery is highly context-dependent, shaped by host developmental stage and baseline immune tone.

### 3.3 Pattern-recognition and inflammasome signaling

The downstream effectors of microbial sensing—TLRs, NOD-like receptors and inflammasomes—are modulated after bariatric surgery in a procedure- and tissue-specific manner. In models of high-fat-diet-driven colorectal cancer, inactivation of the TLR4/MyD88 axis through gut microbiota and bile acid remodeling recapitulates the protective phenotype of SG or RYGB (41). Conversely, in chemotherapy-induced intestinal injury, secondary bile acids that accumulate after bariatric surgery exert context-dependent effects on the NLRP3 inflammasome: they activate NLRP3 under non-inflammatory conditions but suppress it once inflammation is established, illustrating the bidirectional relationship between bile acid signaling and innate effector pathways (42).

Pattern-recognition receptors also mediate the innate sensing of microbial metabolites that accumulate after surgery, including SCFAs, indole derivatives and secondary bile acids, which act on intestinal macrophages, dendritic cells and ILCs to enforce or relax mucosal tolerance (9, 12). A recurrent finding across studies is that the magnitude and direction of innate reprogramming after bariatric surgery are highly contingent on the residual microbiota, on dietary context and on host genetics, which complicates the translation of these mechanisms into predictable biomarkers (3, 4). The net functional outcome depends on the interplay between residual gut taxa, dietary inputs, and host genetic background. Understanding these signaling networks is therefore critical for isolating specific immunomodulatory nodes for non-surgical therapeutic targeting.

## 4 Bile acid–microbiota–innate immune crosstalk

### 4.1 Bile acid pool remodeling after bariatric surgery

Bile acids are increasingly recognized as central signaling molecules that couple the post-surgical microbiota to the innate immune system. RYGB and SG both elevate circulating and tissue bile acid pools, with RYGB producing a more pronounced shift towards secondary, conjugated species (23, 43). The procedure alters the anatomical sites at which bile acids meet the microbiota: in RYGB, bile encounters ingested nutrients only after the alimentary and biliary limbs join, whereas in SG the bile flow pattern is largely preserved but accelerated (4). These anatomical changes drive distinct bile acid profiles, with SADI-S and SG producing unique taurine- and glycine-conjugated bile acid signatures that link to improved glucose metabolism via microbiota-dependent mechanisms (44, 45).

Functional studies indicate that a small-intestinal bile acid axis acts as a primary driver of microbiota remodeling after bariatric surgery: supplementation with a single bile acid can reproduce several of the metabolic improvements observed after SG, suggesting that the bile acid signal is upstream rather than downstream of the microbial changes (4, 46). In cholecystectomy, which mimics some features of the post-bariatric bile flow, secondary bile acids accumulate and ameliorate colitis through inhibition of monocyte/macrophage recruitment, further supporting a direct bile acid–innate immune link (47, 48). These findings position the altered bile acid pool as a primary driver of systemic immune re-education.

### 4.2 FXR and TGR5 signaling in innate immune cells

The farnesoid X receptor (FXR) and the Takeda G protein-coupled receptor 5 (TGR5/GPBAR1) are the two dominant bile acid receptors that couple bile acid signals to innate immune cell function (49, 50). In myeloid cells, FXR agonism suppresses pro-inflammatory cytokine production, particularly IL-1β and TNF-α, by liver and intestinal macrophages, and FXR deficiency in gut macrophages worsens colitis-associated tumourigenesis (51, 52). TGR5, in turn, dampens nuclear factor κB signaling via cAMP–protein kinase A in dendritic cells and skews macrophages towards an anti-inflammatory or even pro-resolving phenotype; TGR5 activation also restrains M2 polarisation in non-small cell lung cancer, indicating that the bile acid–TGR5 axis has direct implications for tumour-associated myeloid cells (53–55).

Bile acids can act as endogenous ligands for mitofusin 2, differentially modulating innate immunity in cholestatic versus physiological conditions (56). At supraphysiological concentrations they promote mitochondrial-endoplasmic reticulum tethering, calcium overload and NLRP3 inflammasome activation, whereas at low concentrations they enhance oxidative phosphorylation and phagocytic clearance of bacteria (56). These concentration-dependent effects help to explain the divergent immunological outcomes reported after bariatric surgery, in which both pro- and anti-inflammatory bile acid-mediated effects have been observed (10, 42). This concentration-dependent duality explains the variable inflammatory responses observed across different anatomical compartments after surgery. At the subcellular level, these shifts converge on key pattern-recognition receptors to suppress pro-inflammatory cascades while priming myeloid cells for anti-tumor effector functions (**Figure 2**).

**Figure 2:**
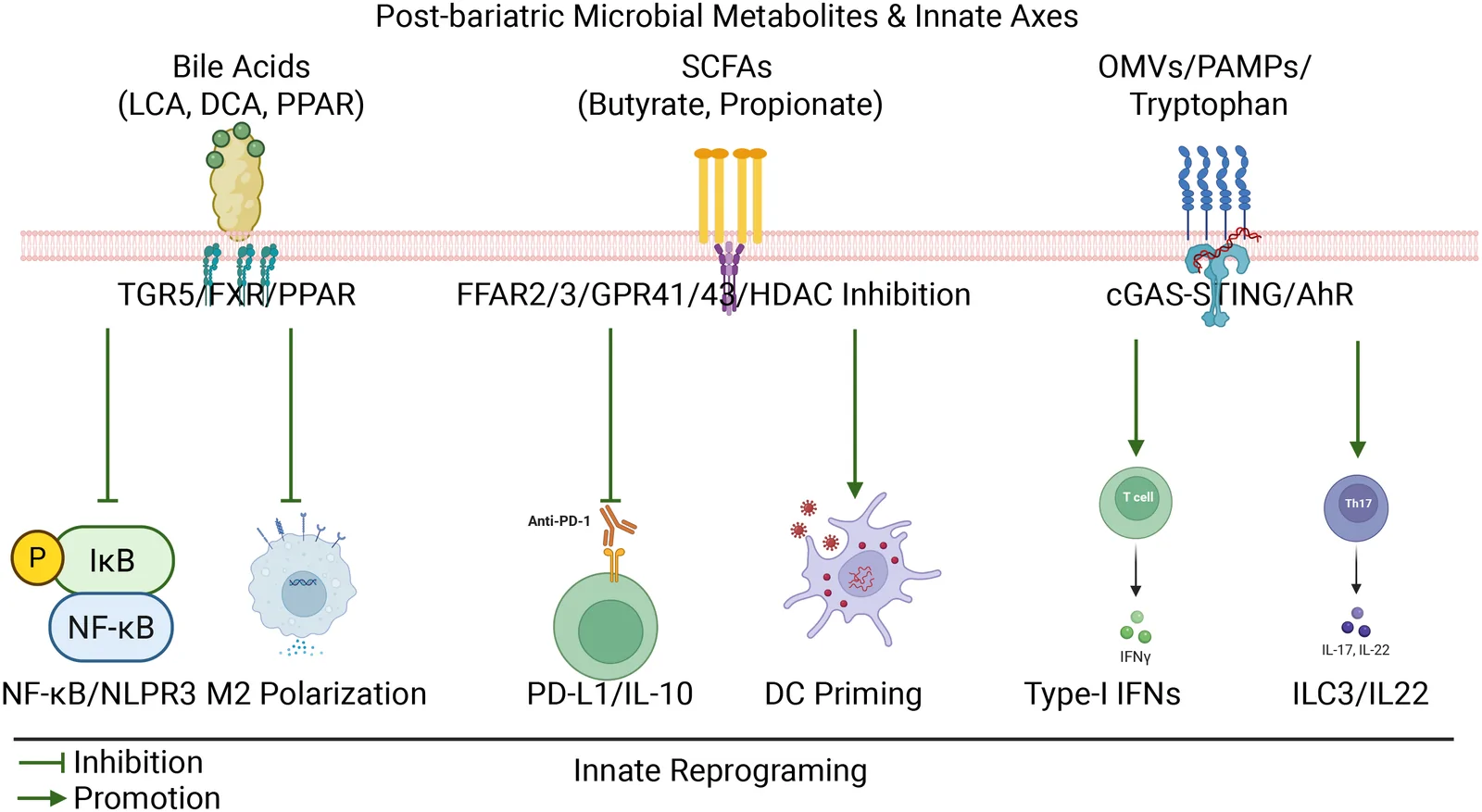
Molecular networks linking post-bariatric microbial metabolites and PAMPs to innate immune cell reprogramming. LCA, lithocholic acid; DCA, deoxycholic acid; PPAR, peroxisome proliferator-activated receptor; SCFAs, short-chain fatty acids; OMVs, outer membrane vesicles; PAMPs, pathogen-associated molecular patterns; TGR5, Takeda G protein-coupled receptor 5; FXR, farnesoid X receptor; FFAR2/3, free fatty acid receptor 2/3; GPR41/43, G protein-coupled receptor 41/43; HDAC, histone deacetylase; cGAS-STING, cyclic GMP-AMP synthase–stimulator of interferon genes; AhR, aryl hydrocarbon receptor; NF-κB, nuclear factor kappa B; NLRP3, NOD-like receptor family pyrin domain containing 3; PD-L1, programed death-ligand 1; IL-10, interleukin-10; DC, dendritic cell; IFN, interferon; ILC3, group 3 innate lymphoid cell; IL-22, interleukin-22.

### 4.3 Bile acids as immunomodulators in the cancer context

In tumor settings, individual bile acid species can either restrain or promote malignancy through innate immune mechanisms. Lithocholic acid (LCA) inhibits dendritic cell maturation via TGR5 and reprograms macrophages towards a pro-resolving state that enhances responsiveness to integrin blockade in Crohn’s disease, demonstrating that a single microbial bile acid can predict therapeutic response (54, 57). Conversely, deoxycholic acid (DCA) cooperates with bacterial lipoteichoic acid to drive pro-tumourigenic M2 macrophage polarisation in obesity-associated hepatocellular carcinoma via TGR5/STAT3 and TLR2/NF-κB signaling, while ursodeoxycholic acid (UDCA) produced by Parabacteroides goldsteinii programes macrophages through PPARγ/GPR132 to ameliorate chronic inflammatory disease (58, 59). Hyodeoxycholic acid (HDCA), produced by Ruminococcus callidus, reshapes intestinal macrophages and limits inflammation through PPARγ-driven fatty acid oxidation (60, 61). Together, these findings argue that bile acid chemistry—shaped strongly by bariatric surgery—should be viewed as a tunable axis for innate immune reprogramming in GI cancers (9, 10). These distinct bioactivities highlight the post-bariatric bile acid pool as a highly tunable chemical library for custom myeloid reprogramming in GI malignancies. It is important to note, however, that secondary bile acids also exert context-dependent and potentially pro-inflammatory or oncogenic effects. DCA, for instance, promotes epithelial barrier disruption, DNA damage, and pro-tumourigenic signaling in the colon, while LCA at elevated concentrations can impair T cell function and suppress HLA class I expression (62–64). These dualistic actions underscore that the immunomodulatory impact of any given bile acid is shaped by its local concentration, the tissue microenvironment, and the prevailing inflammatory tone.

## 5 Microbial metabolites, PAMPs, and obesity-associated dysbiosis

### 5.1 Short-chain fatty acids

Short-chain fatty acids—particularly butyrate, propionate and acetate—are the most extensively studied microbially derived metabolites in the context of innate immunity. In patients undergoing bariatric surgery, post-operative enrichment of SCFA-producing taxa is associated with improved glycaemic control, increased GLP-1 and PYY secretion and reduced systemic inflammation, particularly when reinforced by dietary fibre supplementation (65, 66). In experimental models, butyrate directly suppresses the immunosuppressive factors PD-L1 and IL-10 in tumour-associated macrophages of gastric cancer and inhibits M2 macrophage polarisation in colorectal cancer, providing a mechanistic bridge between microbial metabolites and the cancer immune microenvironment (67, 68).

SCFAs also act on innate lymphoid cells, NK cells and dendritic cells to reinforce mucosal defence. In hepatocellular carcinoma, butyrate enhances NK cell infiltration through CXCL11-dependent signaling, while in colitis-associated colorectal cancer butyrate–macrophage crosstalk restrains M2 polarisation and IL-17-driven inflammation (69, 70). *In vitro* fermentation models confirm that dietary fibre fermentation products shift macrophage-colonocyte interactions in favor of an anti-tumor phenotype, supporting the rationale for fibre- or SCFA-based adjuncts in GI cancer immunotherapy (71). These mechanistic insights support integrating fiber or SCFA supplementation as metabolic adjuncts during cancer immunotherapy.

### 5.2 Tryptophan and other microbial metabolites

Tryptophan catabolites generated by gut bacteria—including indole-3-aldehyde, indole-3-propionic acid, kynurenine and related aryl hydrocarbon receptor (AhR) ligands—are powerful modulators of innate immunity and mucosal homeostasis (1, 72). Kynurenine produced under stress-induced dysbiosis has been shown to remodel tumour-associated macrophages and promote gastric cancer metastasis, suggesting that the microbial tryptophan balance is directly tumour-relevant (73). Conversely, indole metabolites can reinforce intestinal barrier function and AhR signaling in ILC3s, limiting inflammation and improving responsiveness to immune checkpoint blockade (10, 74).

Trimethylamine N-oxide (TMAO), a microbiota-derived metabolite linked to high-protein diets, drives type I interferon signaling in tumour-associated macrophages and boosts responsiveness to immune checkpoint blockade in pancreatic ductal adenocarcinoma, demonstrating that even classically ‘cardiovascular’ microbial metabolites can be harnessed for anti-tumor immunity (75). Conversely, methylglyoxal from gut microbes potentiates radio-immunotherapy in rectal cancer by triggering endoplasmic reticulum stress and cGAS-STING activation, providing another example of how the metabolic output of the microbiota can directly engage innate sensors in the tumor microenvironment (76). Thus, a broad spectrum of post-bariatric metabolites can be directly harnessed to re-arm the host innate immune machinery against solid tumors.

### 5.3 Pathogen-associated molecular patterns and outer membrane vesicles

Bacterial outer membrane vesicles (OMVs), lipopolysaccharide, lipoteichoic acid and microbial DNA constitute the major pathogen-associated molecular patterns (PAMPs) that drive innate sensing through TLR2, TLR4, TLR9 and cytosolic sensors such as cGAS-STING (31, 72). The balance of these PAMPs is shifted by bariatric surgery: enrichment of Akkermansia, Bifidobacterium and Faecalibacterium reduces LPS load and TLR4 signaling, while expansion of oral-associated taxa in the distal gut and altered archaeal communities introduce new microbial products that re-shape innate recognition (15, 77, 78). The net effect is a re-balanced—rather than uniformly attenuated—innate sensing landscape that can be exploited therapeutically in GI cancers (1, 79).

### 5.4 Obesity-associated dysbiosis and gastrointestinal cancer risk

Obesity is associated with an increased incidence and worse outcomes of multiple GI malignancies, and the gut microbiome is increasingly implicated as a mechanistic link (1, 10). Dysbiosis in obese individuals—characterized by reduced alpha-diversity, expansion of pro-inflammatory Proteobacteria and contraction of SCFA-producing Firmicutes—promotes a low-grade endotoxaemia, M2 macrophage polarisation and increased production of IL-6, TNF-α and IL-17 (31, 80). In murine models, high-fat-diet-driven colorectal tumourigenesis is associated with CD206^+^ M2 macrophage infiltration that correlates with the abundance of Bacteroides massiliensis and Vampirovibrion, while in gastric cancer chronic stress-induced enrichment of Lactobacillus reuteri and kynurenine remodels tumour-associated macrophages towards pro-metastatic states (73, 80).

Hepatocellular carcinoma and pancreatic ductal adenocarcinoma are similarly shaped by obesity-induced dysbiosis and innate immune rewiring. Bile acid–TLR crosstalk and obesity-associated secondary bile acid accumulation promote hepatocellular carcinoma progression in mice, while intratumoural bacteria such as Blautia and its metabolite 6-hydroxyhexanoic acid reprogram tumour-associated macrophages towards an anti-tumor phenotype in PDAC (58, 81). Although obesity itself cannot be considered a direct microbial mediator, the obese microbiome is now recognized as a key effector of obesity-associated cancer risk (1, 10). These findings establish the obese microbiome as an active driver of GI cancer risk—and its surgical correction as a logical strategy for immune restoration.

## 6 Gut microbiota and immune checkpoint efficacy in gastrointestinal cancers

### 6.1 Hepatocellular carcinoma

Patients with hepatocellular carcinoma who respond to anti-PD-1 therapy are enriched in gut microbial taxa that support dendritic cell priming and type I interferon signaling, while non-responders display a dysbiotic profile characterized by reduced alpha-diversity and an expansion of pro-inflammatory Proteobacteria (7, 82). A phase II trial combining nivolumab with the PDE5 inhibitor tadalafil and oral vancomycin in patients with refractory HCC, colorectal liver metastases or pancreatic cancer demonstrated measurable reductions in secondary bile acids and shifts in gut microbiota composition alongside stable disease in a small subset of heavily pre-treated patients (83). Mechanistic studies suggest that microbe-derived metabolites such as TMAO can potentiate the type I interferon pathway in tumour-associated macrophages and thereby enhance ICI efficacy, while butyrate and lithocholic acid derivatives can remodel the tumor immune microenvironment through CXCL11-dependent NK cell infiltration (69, 75).

Additional evidence links the cholecystohepatic shunt and microbial secondary bile acids to NKT cell-mediated anti-HCC immunity, indicating that bile acid-targeted interventions may act in concert with ICIs to improve outcomes in liver cancer (48). Recent work further identifies lithocholic acid as an activator of vitamin D receptor signaling in macrophages that promotes HCC recurrence after incomplete thermal ablation, underscoring the complexity of bile acid–innate immune interactions in the liver (84). These nuanced responses underscore the necessity of precisely tailoring bile acid-targeted interventions when combining them with ICIs in liver cancer.

### 6.2 Colorectal cancer

Colorectal cancer provides the strongest evidence for microbiome-mediated modulation of immune checkpoint efficacy, but the picture is heterogeneous. In mismatch-repair-deficient (dMMR) CRC, response rates to anti-PD-1 therapy are high, and the gut microbiome contributes to the baseline inflamed tumor microenvironment; in microsatellite-stable (MSS) disease, which accounts for the majority of metastatic CRC, response rates remain low but can be improved by microbiome-targeted interventions (8, 85). Beneficial gut microbes such as Akkermansia muciniphila, Bacteroides fragilis, Bacteroides uniformis and SCFA producers prime dendritic cells to produce IL-12, polarise Th1 responses and reinvigorate CD8^+^ T cells, whereas Fusobacterium nucleatum and antibiotic-driven dysbiosis foster myeloid suppression and β-catenin- or IL-17-driven resistance to checkpoint blockade (8, 86, 87).

Responder-derived microbial consortia, including the four-strain UJ-04 consortium and Lactobacillus rhamnosus GG, can restore anti-PD-1 efficacy in murine models, and L-arginine-, inosine- and riboflavin-producing taxa are associated with durable clinical benefit in multi-omics analyses (88, 89). Combination strategies that pair intratumoural microbiota-targeted nanoparticles with ICIs, or that combine TLR9 agonism (pixatimod) with nivolumab in MSS metastatic CRC and PDAC, are currently being evaluated and have shown acceptable safety with signals of immune activation (90). The local tumor microenvironment, particularly the abundance of dendritic cells and macrophages, is a critical determinant of whether the gut microbiota signal can be translated into productive anti-tumor immunity (91, 92). Modulating the local myeloid microenvironment thus represents a viable strategy for breaking primary resistance to checkpoint blockade.

### 6.3 Gastric, esophageal and pancreatic cancer

In gastric cancer (GC), the intratumoural microbiota is enriched for Lactobacillus, Ligilactobacillus salivarius and related taxa, and bacterial vesicles from intratumoural L. salivarius can enhance anti-PD-1 blockade through FPR1-mediated macrophage polarisation (93). Butyrate-producing taxa reduce PD-L1 and IL-10 in tumour-associated macrophages, supporting a pro-immunogenic tumor microenvironment that is responsive to checkpoint inhibition (67). Single-cell analyses have further delineated macrophage subtypes with differential responsiveness to ICI in gastric and esophageal cancers, providing potential biomarkers for treatment selection (94, 95).

Pancreatic ductal adenocarcinoma has historically been refractory to immune checkpoint blockade, but microbiome-targeted interventions can overcome some of this resistance. TMAO-driven type I interferon activation, Blautia-derived 6-hydroxyhexanoic acid-mediated macrophage reprogramming and Lactobacillus rhamnosus GG–driven cGAS-STING activation all converge on innate immune engagement that re-sensitises PDAC to ICIs (75, 81, 89). Multi-omics analyses of intratumoural microbiomes in PDAC have identified subtypes with distinct prognosis and immunotherapy responsiveness, providing an actionable framework for patient stratification (96). Together, these studies argue that the gut and intratumoural microbiota are tractable levers for improving ICI responses across GI cancers (12, 79). The immunomodulatory consequences of post-bariatric microbiota reconfiguration manifest through distinct organ-specific mechanisms across gastrointestinal malignancies (**Figure 3**). While in HCC, bile acid-mediated CXCL11 release primarily drives NK and NKT cell recruitment, in PDAC and MSS-CRC, intratumoral microbial vesicles and SCFA-driven DC priming reactivate STING signaling to ignite an otherwise ‘cold’ tumor immune microenvironment.

**Figure 3:**
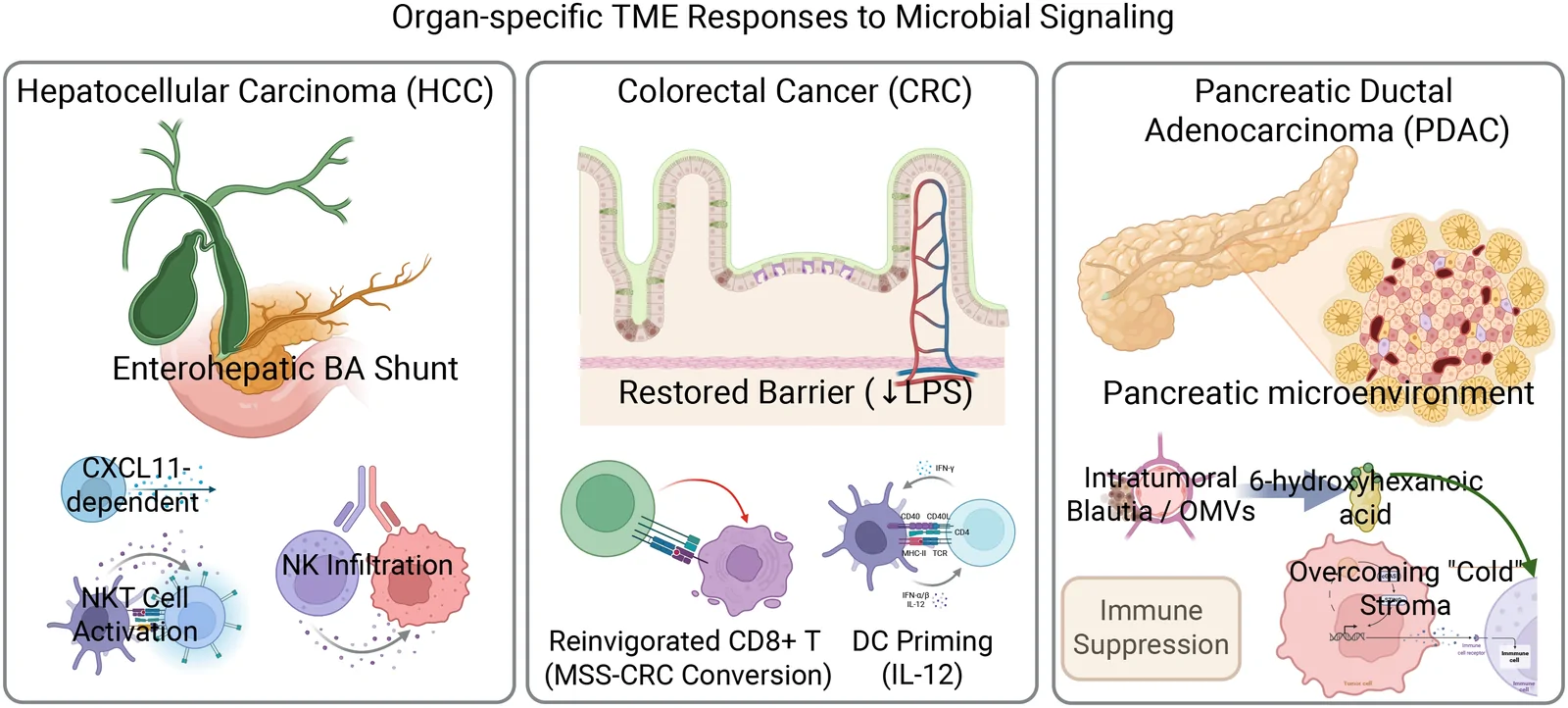
Organ-specific microbial-innate immune crosstalk in gastrointestinal tumor microenvironments. TME, tumor microenvironment; HCC, hepatocellular carcinoma; CRC, colorectal cancer; PDAC, pancreatic ductal adenocarcinoma; BA, bile acid; LPS, lipopolysaccharide; OMVs, outer membrane vesicles; NKT, natural killer T; DC, dendritic cell; IL-12, interleukin-12; STING, stimulator of interferon genes; IFN, interferon; CXCL11, C-X-C motif chemokine ligand 11; NK, natural killer; MSS-CRC, microsatellite-stable colorectal cancer.

## 7 Innate immune pathways and non-surgical translational adjuncts

### 7.1 Macrophage polarization and innate sensors in tumor immunotherapy

Tumour-associated macrophages (TAMs) are the dominant innate effector cells that translate microbiota-derived signals into ICI responsiveness. Across GI cancers, M2-like TAMs correlate with resistance to anti-PD-1 therapy, while M1-polarised or reprogramed macrophages are associated with durable responses (92, 94, 97). Microbial metabolites such as butyrate, indole derivatives, deoxycholic acid and lithocholic acid directly modulate macrophage polarisation through FXR, TGR5, TLR4/MD2, NLRP3 and AhR signaling, providing multiple actionable nodes (61, 67, 68). Microbiota-dependent regulation of costimulatory and coinhibitory pathways via innate sensors—including CD200R1 engagement by the gut microbiota protein BOC1—further illustrates that microbial ligands can mimic or potentiate ICI biology (98, 99).

TREM2 on intestinal macrophages has emerged as a key negative regulator of anti-PD-1 efficacy, and TREM2 deficiency expands Ruminococcus gnavus in the gut and TNF-producing CD4^+^ T cells in the tumor bed, identifying a tractable myeloid-microbiota axis for therapeutic intervention (100). Conversely, innate co-stimulatory pathways in DCs and ILCs support mucosal and systemic anti-tumor immunity, and gut microbial modulation can be harnessed to reinforce these signals (99, 101). Target-specific tuning of myeloid signaling pathways therefore provides a powerful lever for optimizing immunotherapeutic outcomes.

### 7.2 cGAS-STING activation and PRR agonism as therapeutic adjuncts

The cGAS-STING pathway links cytosolic DNA sensing to type I interferon production and is increasingly recognized as a mediator of microbiota-driven anti-tumor immunity. In pancreatic cancer models, the live biotherapeutic Enterococcus lactis MNC-168 releases bacterial membrane vesicles that activate STING in tumor tissues and potentiate anti-PD-1 therapy (102). Lactobacillus rhamnosus GG induces STING expression in tumor lesions, and a STING-targeted PET tracer ([^18^F]FBTA) has been developed to track early microbiota-induced tumor immune remodeling and guide immunotherapy (89). Iron-based single-atom nanozymes can clear tumor-resident intracellular microbiota and potentiate immunotherapy through NOX-like, POD-like and GSHOx-like activities coupled to STING activation (103). These findings position the STING axis as a central integrator of microbial signals and innate immune activation in GI cancer immunotherapy (79, 104).

Innate DNA and RNA sensors also mediate the anti-tumor effects of microbial metabolites such as methylglyoxal, which triggers endoplasmic reticulum stress and cGAS-STING activation to enhance radio-immunotherapy in rectal cancer (76). The convergence of dietary, microbial and metabolic signals on STING provides a unifying mechanistic framework for microbiota-driven innate immune activation in GI cancers (12, 72).

Because pattern-recognition receptors (PRRs) are the primary sensors of microbial signals, pharmacological PRR agonism has emerged as a strategy to mimic or amplify the microbiota-immune crosstalk. The TLR9 agonist pixatimod in combination with nivolumab showed acceptable safety and pharmacodynamic activity in MSS metastatic colorectal cancer and PDAC, while TLR7 agonism combined with CD47-SIRPα blockade produced robust anti-tumor immunity in CT26 colon tumor models (105, 106). TLR9-based neoantigen vaccines using CpG-adjuvanted Adpgk peptides and nano-formulated CpG oligodeoxynucleotides induce potent anti-tumor T cell responses in CRC, and lipid nanoparticle-armoured Limosilactobacillus reuteri combines innate immune stimulation with photo-immunotherapy in colorectal cancer (107, 108).

Combination strategies that pair TLR or STING agonism with ICIs are particularly attractive in MSS CRC and PDAC, where response rates to checkpoint blockade alone remain low (8, 90). The combination of innate agonism with FMT or defined probiotic consortia (e.g., UJ-04, L. salivarius CCFM 1266) further integrates the principles of microbiota-centered and innate-centered immunomodulation and is currently being explored in early-phase clinical trials (88, 109).

### 7.3 Evidence from bariatric surgery to microbiome-targeted clinical trials

A landmark preclinical study demonstrated that the gut microbiome of formerly obese mice that had undergone bariatric surgery enhanced the response to αPD-1 immunotherapy in a model of breast cancer, and that FMT from post-bariatric patients reduced tumor burden and doubled the effectiveness of checkpoint blockade in recipient mice, identifying branched-chain amino acids (BCAAs) as one of the operative microbially derived metabolites (6). These findings extend earlier evidence that obesity-associated dysbiosis impairs ICI efficacy and suggest that durable weight loss and microbiota reconfiguration can convert an immunologically cold tumor microenvironment into an ICI-responsive one (1, 8).

The corollary is that post-bariatric patients may be a population in which ICI responsiveness is enhanced, although this hypothesis requires prospective validation. Additional evidence shows that bariatric surgery is associated with long-term reductions in obesity-associated cancer risk, and pre-clinical VSG models have been developed to investigate how surgery-induced weight loss mediates cancer protection, providing a translational platform for studying the immune consequences of surgery in the cancer setting (110, 111).

### 7.4 Metabolic–immune reprogramming as a therapeutic axis

Bariatric surgery illustrates that durable reconfiguration of metabolic, microbial and innate immune circuits is achievable and can be translated into clinically meaningful benefits. Microbiota-derived metabolites such as butyrate, secondary bile acids, indole derivatives and TMAO engage distinct innate receptors and signaling pathways, providing a portfolio of candidate adjuncts that can be deployed in combination with ICIs (12, 72). Anti-inflammatory macrophage reprogramming through bile acid–TGR5 signaling, STING activation by microbial vesicles, and PRR agonism are emerging as tractable therapeutic nodes that mirror the effects of bariatric surgery without the surgical intervention itself (89, 102, 105).

Exercise and dietary interventions, including dietary fibre and resistant starch supplementation, recapitulate some of the microbiota-related benefits of bariatric surgery and may serve as safer adjuncts to ICI regimens in patients who are not surgical candidates (66, 112). Integrating such interventions with ICI therapy will require careful monitoring of the gut barrier, the mycobiome, virome and archaeome, all of which are altered after bariatric surgery and may contribute to the durability of the immune-metabolic response (77, 113).

### 7.5 Microbiome-targeted strategies in trial design

Clinical trials in GI cancer immunotherapy are increasingly incorporating microbiome endpoints. FMT has shown efficacy in refractory immune checkpoint inhibitor colitis, with first-line FMT achieving clinical response in the majority of treated patients and reproducible increases in alpha-diversity and modulation of plasma cell, neutrophil, macrophage and T cell subsets (114). Defined consortia, engineered probiotics and postbiotics such as heat-killed Latilactobacillus and outer membrane vesicle-based adjuvants are under evaluation to enhance ICI efficacy while reducing immune-related adverse events (74, 88, 114).

Standardisation of microbiome analyses, including 16S rRNA sequencing, shotgun metagenomics, metabolomics and single-cell host transcriptomics, will be essential to identify predictive biomarkers and reproducible therapeutic targets (7, 85). Bariatric surgery provides a uniquely informative natural experiment for prioritising these biomarkers and for testing whether durable microbiota reconfiguration can be recapitulated by less invasive interventions in GI cancer patients (3, 6). Translating the natural experiment of bariatric surgery into non-invasive cancer therapeutics requires a multi-pronged approach (**Supplementary Figure S1**). By combining multi-omics biomarker profiling with synthetic microbial consortia, postbiotic OMVs, and pharmacological PRR/STING agonists, ongoing clinical trial designs aim to mimic post-bariatric innate immune activation to convert ICI-resistant GI cancers into durable responders.

## 8 Challenges, future perspectives, and conclusion

Several challenges must be addressed before the insights from bariatric surgery can be systematically translated into GI cancer immunotherapy. First, the heterogeneity of microbial and immunological responses to bariatric surgery is high, and reproducible predictive biomarkers that distinguish responders from non-responders are still lacking (25, 27). Second, the post-surgical microbiome is shaped by anatomical reconfiguration rather than a uniform set of microbial signals, which complicates the design of trials that aim to mimic surgery-induced changes pharmacologically (4, 44). Third, innate immune activation after bariatric surgery is context dependent: the same bile acid species or microbial metabolite can produce opposing immunological effects depending on the inflammatory milieu and the tissue examined (42, 56). Fourth, the durability of the microbiota and innate immune changes after bariatric surgery is not fully characterized, and the contribution of the mycobiome, virome and archaeome to the immune phenotype is still emerging (77, 113). Finally, prospective clinical trials are needed to determine whether bariatric surgery or its surrogate interventions can enhance ICI efficacy in patients with GI cancers, and whether such benefits outweigh the risks of post-operative malnutrition, gastrointestinal symptoms and potential colorectal cancer risk associated with specific procedures (111, 115). Resolving these challenges is key to clinical translation.

Bariatric surgery works partly through anatomy: altered gastric acid, faster transit, and rerouted enterohepatic circulation. These changes create a niche that oral microbes and metabolites cannot easily reproduce. In a prospective trial of a defined microbial consortium in cancer patients undergoing chemoradiation, no engraftment was observed in the overall cohort (116). Exogenous consortia often fail to colonize, and postbiotic metabolites have short half-lives and poor site-specific delivery. PRR agonists carry further risk: systemic TLR or STING activation can cause cytokine release, mucosal damage, and autoimmune flares, especially in patients already receiving ICIs. A phase III trial of a TLR9 agonist combined with chemotherapy in advanced NSCLC was discontinued early because of increased sepsis-related serious adverse events in the investigational arm (117). Bile acid receptor agonists may also worsen cholestasis, diarrhea, or hepatotoxicity at effective doses; FXR agonists such as obeticholic acid are limited by dose-dependent pruritus, and long-term safety remains unclear (118–120). Trials of these surrogates therefore need pharmacokinetic and colonization monitoring, dose-limiting toxicity data, and stepwise escalation in cancer patients before they can be considered practical substitutes for surgery.

Looking ahead, the convergence of microbiome science, innate immunology and cancer immunotherapy is producing a coherent framework in which the gut–host interface is a central determinant of ICI responsiveness. Bariatric surgery demonstrates that durable reconfiguration of this interface is feasible, that it engages defined innate sensors and signaling pathways, and that it can be translated into clinically meaningful outcomes. A logical next step is the design of trials that pair ICI therapy with surgical, dietary or microbiome-targeted interventions that mimic the post-bariatric phenotype, with the goal of converting more GI cancers into ICI-responsive disease (6, 8, 12). This paradigm offers a promising route to enhance immunotherapy efficacy.

Bariatric surgery serves as a powerful human model demonstrating that lasting immunometabolic rewiring is clinically feasible. By restructuring gut anatomy, altering bile acid kinetics, and resolving systemic meta-inflammation, surgical intervention directly engages innate sensors—including FXR, TGR5, AhR, and cGAS-STING—to reprogram immunosuppressive myeloid cells and enhance tumor-infiltrating lymphocyte activity. Translating these post-bariatric insights into precision oncology offers a viable path to overcome immunotherapy resistance. By leveraging synthetic microbial consortia, postbiotic vesicles, and pharmacological PRR agonists alongside immune checkpoint blockades, these non-surgical surrogates hold clear potential to convert refractory gastrointestinal malignancies into durable, treatment-responsive diseases.

## Supplementary material

Supplementary Figure 1 Translational framework: Leveraging post-bariatric microbial-innate immune mechanisms for precision GI cancer immunotherapy. FMT, faecal microbiota transplantation; OMVs, outer membrane vesicles; SCFAs, short-chain fatty acids; TGR5, Takeda G protein-coupled receptor 5; FXR, farnesoid X receptor; TLR9, Toll-like receptor 9; STING, stimulator of interferon genes; GI, gastrointestinal; ICI, immune checkpoint inhibitor.
